# Eat, Sleep, Console model for neonatal opioid withdrawal syndrome: a meta-analysis

**DOI:** 10.3389/fped.2024.1416383

**Published:** 2024-08-16

**Authors:** Liangliang Chu, Xiaoyi Liu, Cuiping Xu

**Affiliations:** ^1^ School of Traditional Chinese Medicine, Shandong University of Traditional Chinese Medicine, Jinan, Shandong, China; ^2^ School of Nursing, Shandong Academy of Medical Sciences, Shandong First Medical University, Taian, Shandong, China; ^3^ Department of Nursing, The First Affiliated Hospital of Shandong First Medical University, Jinan, Shandong, China

**Keywords:** Eat, Sleep, Console model, Finnegan Neonatal Abstinence Scoring System, neonatal opioid withdrawal syndrome, assessment, meta-analysis

## Abstract

**Background:**

The rising incidence of drug abuse among pregnant women has rendered neonatal opioid withdrawal syndrome a significant global health concern.

**Methods:**

Databases including PubMed, Web of Science, the Cochrane Library, Embase, Elton B. Stephens. Company (EBSCO), China National Knowledge Infrastructure (CNKI), and Wanfang were searched for comparative studies of the Eat, Sleep, Console model vs. traditional assessment tools for neonatal opioid withdrawal syndrome. Two reviewers conducted literature searches, screened according to the inclusion criteria, extracted data, and independently verified accuracy. All meta-analyses were conducted using Review Manager Version 5.4.

**Results:**

In total, 18 studies involving 4,639 neonates were included in the meta-analysis. The Eat, Sleep, Console model demonstrated superior outcomes in assessing neonatal opioid withdrawal syndrome, significantly reducing the need for pharmacological treatment [risk ratio = 0.44, 95% confidence interval (CI) = 0.34–0.56, *P *< 0.001], decreasing the length of hospital stay [standard mean difference (SMD) = −2.10, 95% CI = −3.43 to −0.78, *P = *0.002], and shortening the duration of opioid treatment (SMD = −1.33, 95% CI = −2.22 to −0.45, *P = *0.003) compared to the Finnegan Neonatal Abstinence Scoring System.

**Conclusions:**

The Eat, Sleep, Console model is more effective than the Finnegan Neonatal Abstinence Scoring System in improving the assessment and management of neonatal opioid withdrawal syndrome.

## 1 Introduction

Neonatal opioid withdrawal syndrome (NOWS) occurs in over 55% of infants born to mothers who are addicted to or treated with opioids during pregnancy (1, 2). The clinical symptoms of NOWS typically manifest within the first few days post-birth and include gastrointestinal disturbances, irritability, hypertonia, and seizures (2, 3). With the rising prevalence of drug abuse among pregnant women, the severity of NOWS has increased. This syndrome is now a global concern, causing extended hospital stays, heightened demand for drugs, and increased hospitalization costs, particularly in the United States and Canada (1, 4–6), thus exacerbating the economic burden (7). Since 2014, management strategies for NOWS have evolved to include pharmacological and non-pharmacological interventions, as well as streamlined clinical evaluations (8). However, due to regional and institutional variations, between 50% and 80% of affected newborns require pharmacological treatment (9).

In the United States, the Finnegan Neonatal Abstinence Scoring System (FNASS), devised in 1975, remains the predominant method for assessing NOWS (10, 11). The FNASS, which is based on the observed severity of 21 withdrawal symptoms, involves scoring neonates every 2–6 h by pediatricians or nurses, with pharmacological treatment initiated if scores reach ≥12 or if three consecutive scores of ≥8 are recorded (12). Despite the development of new objective scoring methods, the FNASS is still employed in over 52% of cases involving newborns with NOWS (13). To enhance the reliability of the FNASS in clinical settings, healthcare providers must participate in initial and ongoing training (14). However, studies consistently show that the FNASS may lead to unnecessary pharmacological treatments, resulting in prolonged hospital stays. Extended stays, coupled with less-than-ideal conditions, hinder family members from engaging in the care for infants (15, 16).

In 2017, Grossman et al. introduced a novel model for assessing NOWS, termed the Eat, Sleep, Console (ESC) model (15). This model evaluates crucial physiological functions, such as eating and sleeping. Under the ESC model, pharmacological interventions are withheld if these functions remain unaffected, even if FNASS scores exceed 8 (17). Unlike the FNASS, the ESC model promotes family involvement in the care of infants with NOWS and encourages active participation in managing symptoms (18). In addition, by prioritizing non-pharmacological interventions that increase family engagement, both the need for pharmacological treatment and the duration of hospital stays are significantly reduced (19, 20). Although numerous studies have compared the ESC model with the FNASS, no meta-analysis like this one has previously been conducted. This review aims to vividly illustrate the differences between these two assessment tools and determine if the ESC model provides better outcomes for managing NOWS than traditional methods. The findings are expected to offer practical and theoretical support for selecting an evaluation approach for NOWS in the future.

## 2 Methods

### 2.1 Review protocol

Studies utilizing the ESC model for assessing NOWS were reviewed. Exposure to prenatal opioids included heroin, prescription opioids, over-the-counter opioids, and prescription or illegal opioid replacement therapies. Individuals who used multiple substances were excluded from this analysis. The inclusion criteria are listed in Table 1. Pharmacological treatment requirement was designated as the primary outcome, and hospital stay duration and opioid treatment duration were the secondary outcomes.

**Table 1:** Eligibility criteria for review and meta-analysis.

Condition treated	Neonatal abstinence syndrome
Experiment group	ESC model: **Eat**—the newborn should eat an appropriate amount based on days of age. For the 1- to 2-day-old, this may be less than an ounce per feeding. For 3 days old or greater, this should be 1 or more ounce(s) per feed. Breastfeeding quality should be “good” as defined by the mother and nursing staff assessment. **Sleep**—the newborn should be able to sleep undisturbed for a minimum of 1 h. **Console**—the newborn should be consoled within 10 min. If not, non-pharmacologic interventions should be increased including a second caregiver making attempts to console the newborn. If the newborn remains inconsolable, this would be an indication that the newborn may need pharmacologic treatment and the medical team should be notified.
Control group	FNASS
Study design	Before and after studies with allocation to Eat, Sleep, Console model vs. Finnegan Neonatal Abstinence Scoring System for neonatal abstinence syndrome as control
Primary outcome evaluated	Need for pharmacologic treatment
Secondary outcome evaluated	Length of hospital stay; length of opioid treatment stay

### 2.2 Search strategy

This meta-analysis was performed according to the guidelines of Preferred Reporting Items for Meta-Analyses (PRISMA) (21). We searched the PubMed, ScienceNet, Cochrane Library, Embase, Elton B. Stephens. Company (EBSCO), China National Knowledge Infrastructure (CNKI), and Wanfang databases to collect comparative studies of the Eat, Sleep, Console model vs. traditional assessment tools for NOWS up to January 2024, using the following words: prenatal opioid exposure OR neonatal passive addiction OR neonatal opioid withdrawal syndrome OR neonatal withdrawal syndrome OR neonatal opioid withdrawal syndrome, Eat, Sleep, Console model OR ESC, assessment. The retrieval strategy adopted was based on PubMed as an example is as follows:

**1#** “prenatal opioid exposure”[TW] OR “neonatal passive addiction”[TW] OR “neonatal opioid withdrawal”[TW] OR “neonatal opioid withdrawal syndrome”[TW]
**2#** “neonatal abstinence syndrome”[MeSH] OR “neonatal abstinence syndrome”[TW] OR “neonatal withdrawal syndrome”[TW] OR neonatal[TIAB] OR abstinence[TIAB] OR withdrawal[TIAB]
**3#** 1# OR 2#
**4#** “Infant, Newborn”[Mesh] OR “newborn infant”[TIAB] OR infant[TIAB] OR newborn[TIAB]
**5#** “Analgesics, Opioid”[Mesh] OR “Analgesics, Opioid” [Pharmacological Action] OR “opioid analgesics”[TIAB] OR opioid[TIAB]
**6#** “eat, sleep, console”[TW] OR eat[TW] OR sleep[TW] OR console[TW] OR comfort[TW] OR ESC[TW]
**7#** 3# OR 4# AND 5# AND 6#

### 2.3 Study selection and data collection

The literature searches and screenings were independently conducted by two reviewers. After eliminating duplicates, titles and abstracts were examined. A detailed review of the full texts established eligibility. Discrepancies were resolved through discussion with the corresponding author. Following the selection of studies, detailed data extraction was performed by the reviewers. From the studies included, a matrix was constructed detailing the author's name, year of study, study design, sample characteristics, intervention comparisons, outcomes, and outcome data.

### 2.4 Statistical methods

Statistical analysis was conducted using Review Manager (version 5.4; Cochrane Informatics & Technology Services) software, considering a *P*-value <0.05 as statistically significant. Discontinuous variables were analyzed using the risk ratio (RR) and 95% confidence interval (CI), while continuous outcomes utilized the standard mean difference (SMD) and 95% CI. Heterogeneity was assessed using the *I*^2^ statistic. Values of *I*^2^ between 0% and 40% indicate minimal heterogeneity, while 75%–100% suggest high heterogeneity. Moderate heterogeneity was indicated by *I*^2^ values between 30% and 60%, and significant heterogeneity by 50%–90%. For *I*^2^ ≥50, a random-effects model was applied; for *I*^2^ <50, a fixed-effects model was utilized. Potential sources of heterogeneity, including differences in health education, non-pharmacological treatment methods, literature quality, and gestational age, prompted the use of a random-effects model. In addition, a sensitivity analysis was performed by excluding each study individually to identify the sources of high heterogeneity.

### 2.5 Quality assessment

The quality of included randomized controlled trials was assessed using the Cochrane Handbook's recommended tool. This tool evaluates seven bias-related aspects, including selection, performance, and detection bias, categorizing the risk of bias into three levels: low, high, and unclear (Table 2). The risk of bias in non-randomized studies was assessed using the ROBINS-I tool (23), which evaluates four levels of bias risk: low, moderate, serious, and no information (Table 3). Bias risk assessments were independently conducted by two authors, with discrepancies resolved by consulting the corresponding author.

## 3 Results

### 3.1 Study selection and characteristics

**Figure 1:**
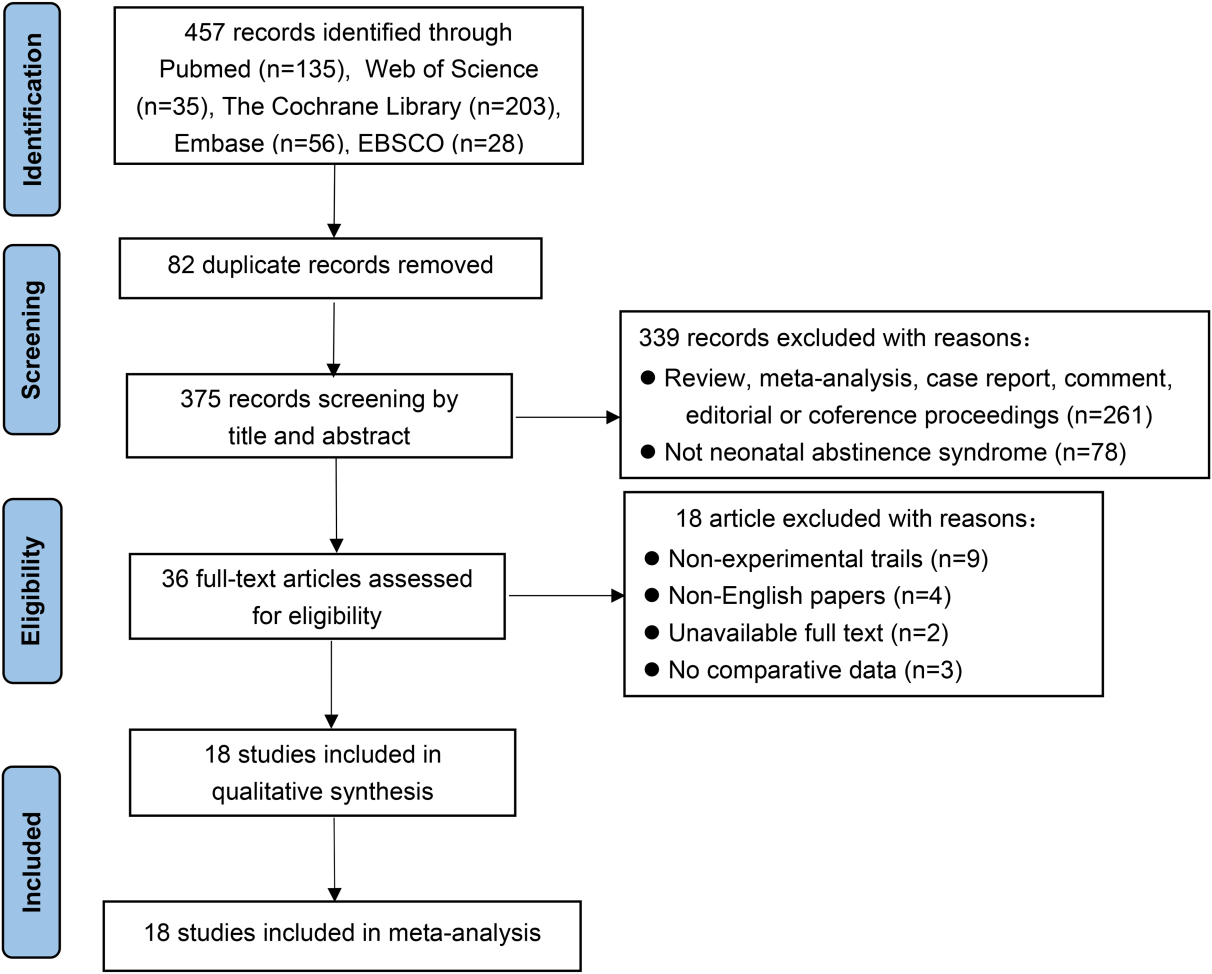
PRISMA flow diagram: papers included and excluded in this review.

Of the 457 studies reviewed, 18 (15, 17, 22, 24–38) were included in this meta-analysis. The literature search process and outcomes are illustrated in Figure 1. A total of 4,639 patients were analyzed, with 2,003 (43.2%) assessed using the ESC model and 2,636 (56.8%) using the FNASS. These studies, conducted between 2017 and 2023, varied in sample size, geographic location, and clinical setting (Table 4).

### 3.2 Requirement for pharmacotherapy

**Figure 2:**
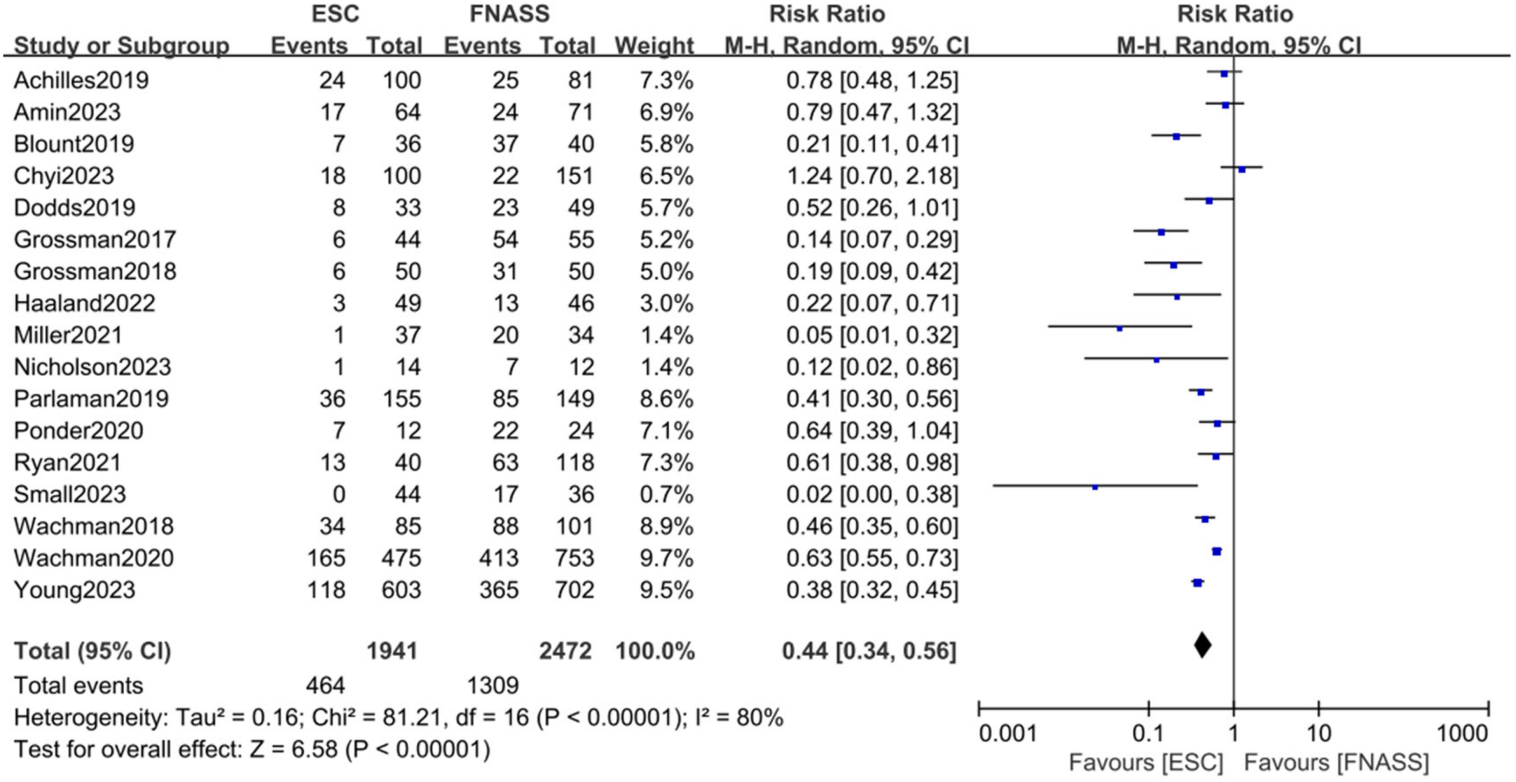
Eat, Sleep, Console model vs. Finnegan Neonatal Abstinence Scoring System on the requirement for pharmacotherapy. ESC, Eat, Sleep, Console model; FNASS, Finnegan Neonatal Abstinence Scoring System; CI, conﬁdence interval; M-H, Mantel–Haenszel.

Seventeen studies (15, 17, 22, 24–36, 38) demonstrated that infants with NOWS were less likely to require pharmacological treatment under the ESC model compared to the FNASS. As depicted in Figure 2, the ESC model showed superior outcomes (RR = 0.44, 95% CI = 0.34–0.56, *P *< 0.001). Significant heterogeneity was noted (*P *< 0.001, *I*^2^* *= 80%), potentially due to variations in health education, non-pharmacological treatment methods, literature quality, and gestational age.

#### 3.2.1 Subgroup analysis

##### 3.2.1.1 Overview

**Figure 3:**
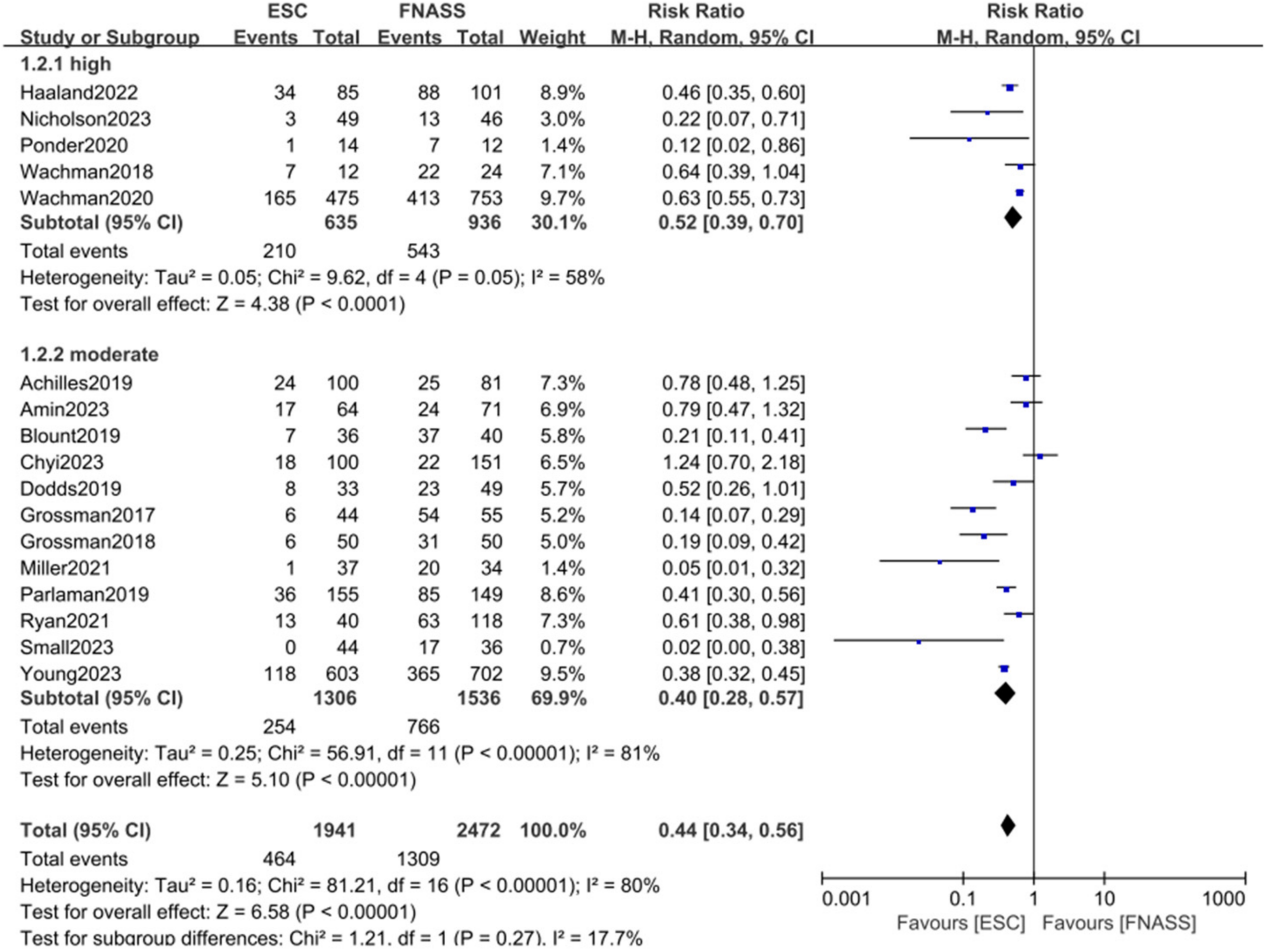
Subgroup analysis on the outcome of assessment categorized by literature quality (group 1: high quality; group 2: moderate quality). ESC, Eat, Sleep, Console model; FNASS, Finnegan Neonatal Abstinence Scoring System; M-H, Mantel–Haenszel; CI, conﬁdence interval.

**Figure 4:**
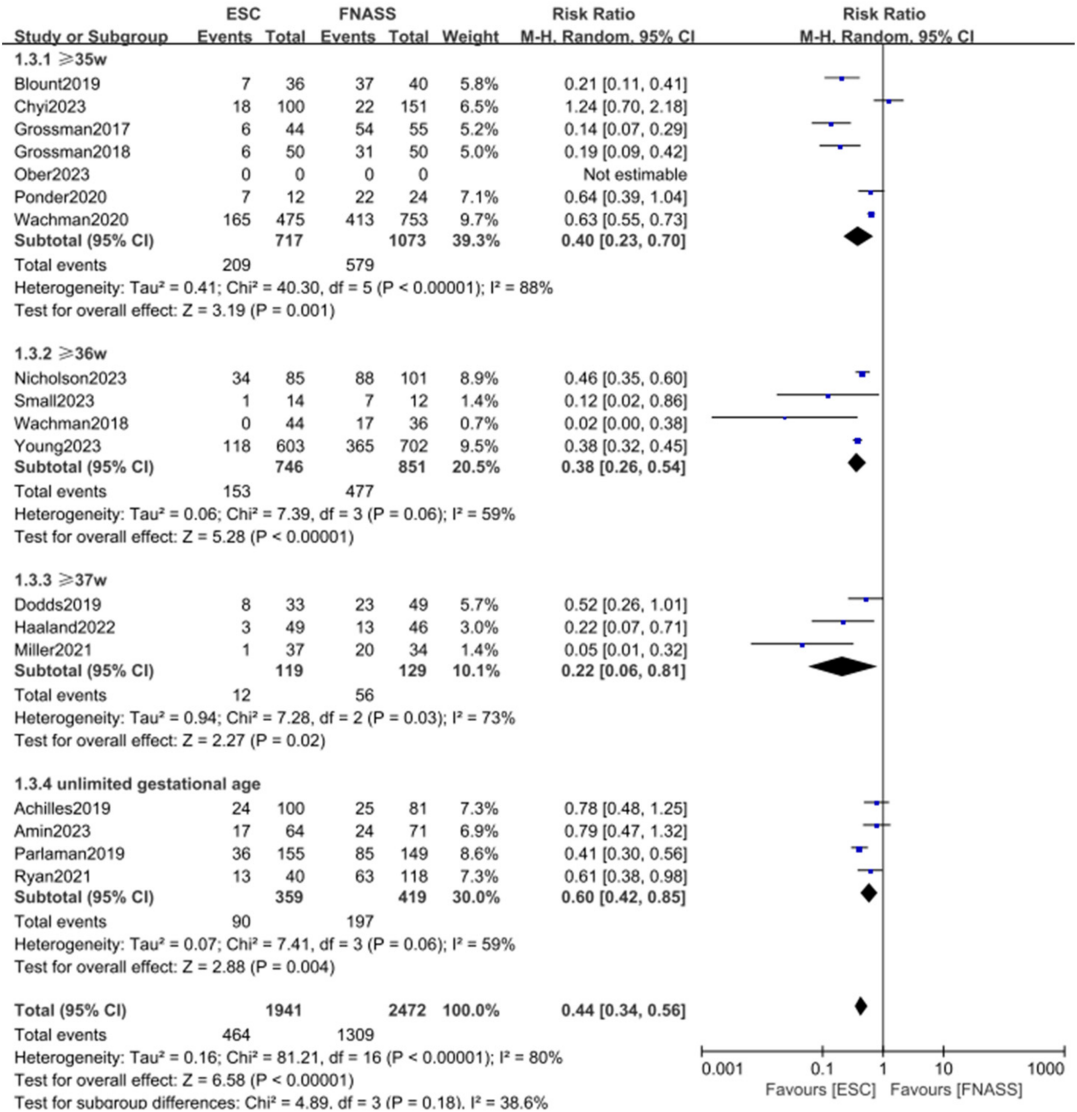
Subgroup analysis on the outcome of assessment categorized by gestational age (group1: ≥35 weeks; group 2: ≥36 weeks; group 3: ≥37 weeks; group 4: unlimited gestational age). ESC, Eat, Sleep, Console model; FNASS, Finnegan Neonatal Abstinence Scoring System; M-H, Mantel–Haenszel; CI, conﬁdence interval.

Health education and non-pharmacological treatment interventions (e.g., demand feeding, promotion of breastfeeding, parental bedside presence, skin-to-skin contact, swaddling, environmental stimulation reduction, and caloric enhancement of formula) are commonly implemented alongside the ESC model. Given that the included studies originated from various hospitals across different regions, notable similarities and differences in the approaches to health education and non-pharmacological interventions were observed. Consequently, subgroup analyses were precluded. Instead, analyses were conducted based on literature quality and gestational age, with results presented in Figures 3, 4.

##### 3.2.1.2 First subgroup categorized by literature quality

The subgroup analysis, based on literature quality, categorized studies into high- and moderate-quality groups. As depicted in Figure 3, outcomes were more favorable in the ESC model (subgroup 1: RR = 0.52, 95% CI = 0.39–0.70; *P *< 0.001; subgroup 2: RR = 0.40, 95% CI = 0.28–0.57; *P *< 0.001). Both groups exhibited some heterogeneity (*P *= 0.05, *I*^2^* *= 58%, vs. *P *< 0.001, *I*^2^* *= 81%), with observable subgroup differences (*P *= 0.27, *I*^2^* *= 17.7%). In addition, the analysis indicated a reduced requirement for pharmacotherapy in the ESC model (RR = 0.44, 95% CI = 0.34–0.56; *P *< 0.001).

##### 3.2.1.3 Second subgroup categorized by gestational age

The second subgroup analysis was based on gestational age, including categories of ≥35 weeks, ≥36 weeks, ≥37 weeks, and undefined gestational age. Figure 4 shows that outcomes were similarly more favorable in the ESC model (subgroup 1: RR = 0.40, 95% CI = 0.23–0.70, *P = *0.001; subgroup 2: RR = 0.38, 95% CI = 0.26–0.54, *P *< 0.001; subgroup 3: RR = 0.22, 95% CI = 0.06–0.81; *P = *0.02; subgroup 4: RR = 0.60, 95% CI = 0.42–0.85; *P = *0.004). All groups displayed considerable heterogeneity (subgroup 1: *P *< 0.001, *I*^2^* *= 88%; subgroup 2: *P = *0.06, *I *^2^ = 59%; subgroup 3: *P *= 0.03, *I*^2^* *= 73%; subgroup 4: *P *= 0.06, *I*^2^* *= 59%), with observed subgroup differences (*P *= 0.18, *I*^2^* *= 38.6%). Moreover, the ESC model was associated with a significant overall benefit (RR = 0.44, 95% CI = 0.34–0.56; *P *< 0.001).

### 3.3 Length of hospital stay

**Figure 5:**
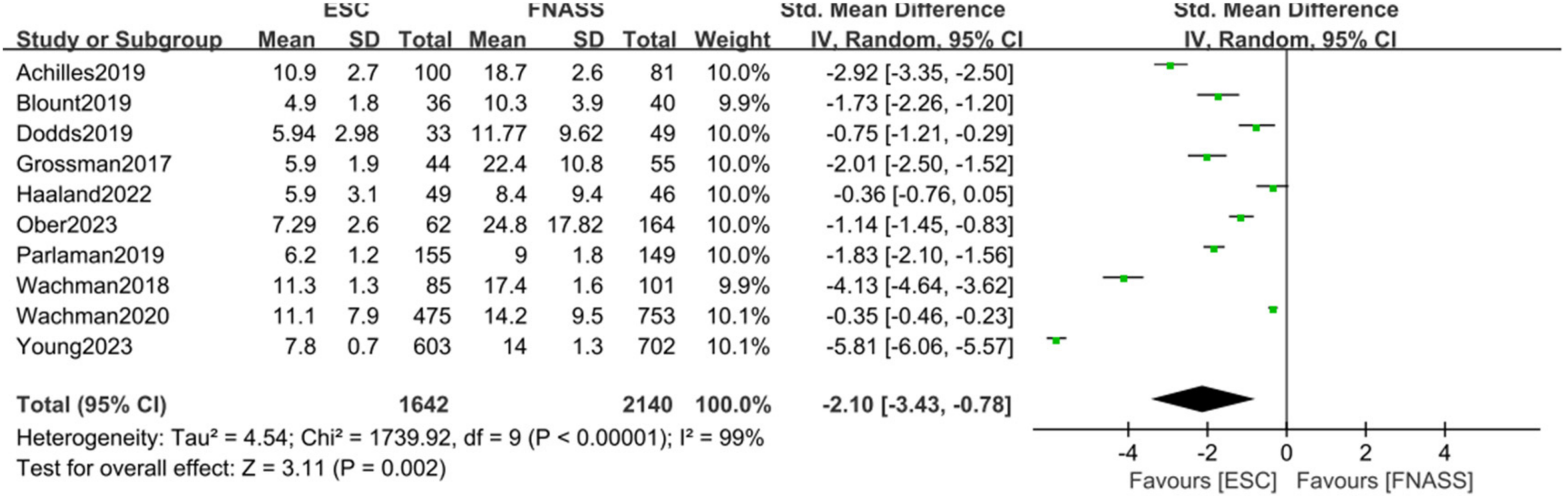
Eat, Sleep, Console model vs. Finnegan Neonatal Abstinence Scoring System on the length of hospital stay. SD, standard deviation; IV, inverse variance; CI, conﬁdence interval; ESC, Eat, Sleep, Console model; FNASS, Finnegan Neonatal Abstinence Scoring System.

Ten studies (15, 22, 24–28, 30, 33, 37) found that infants with NOWS who underwent ESC model assessment experienced significantly shorter hospital stays compared to those who underwent FNASS assessment. According to the meta-analysis presented in Figure 5, the ESC model yielded better outcomes (SMD = −2.10, 95% CI = −3.43 to −0.78, *P = *0.002). Significant heterogeneity was observed (*P *< 0.001, *I*^2^ = 99%), potentially due to variations in health education and non-drug treatment interventions. A sensitivity analysis was conducted by excluding each study individually, revealing no substantial changes in the results, which affirmed the reliability of the meta-analysis.

### 3.4 Length of opioid treatment stay

**Figure 6:**
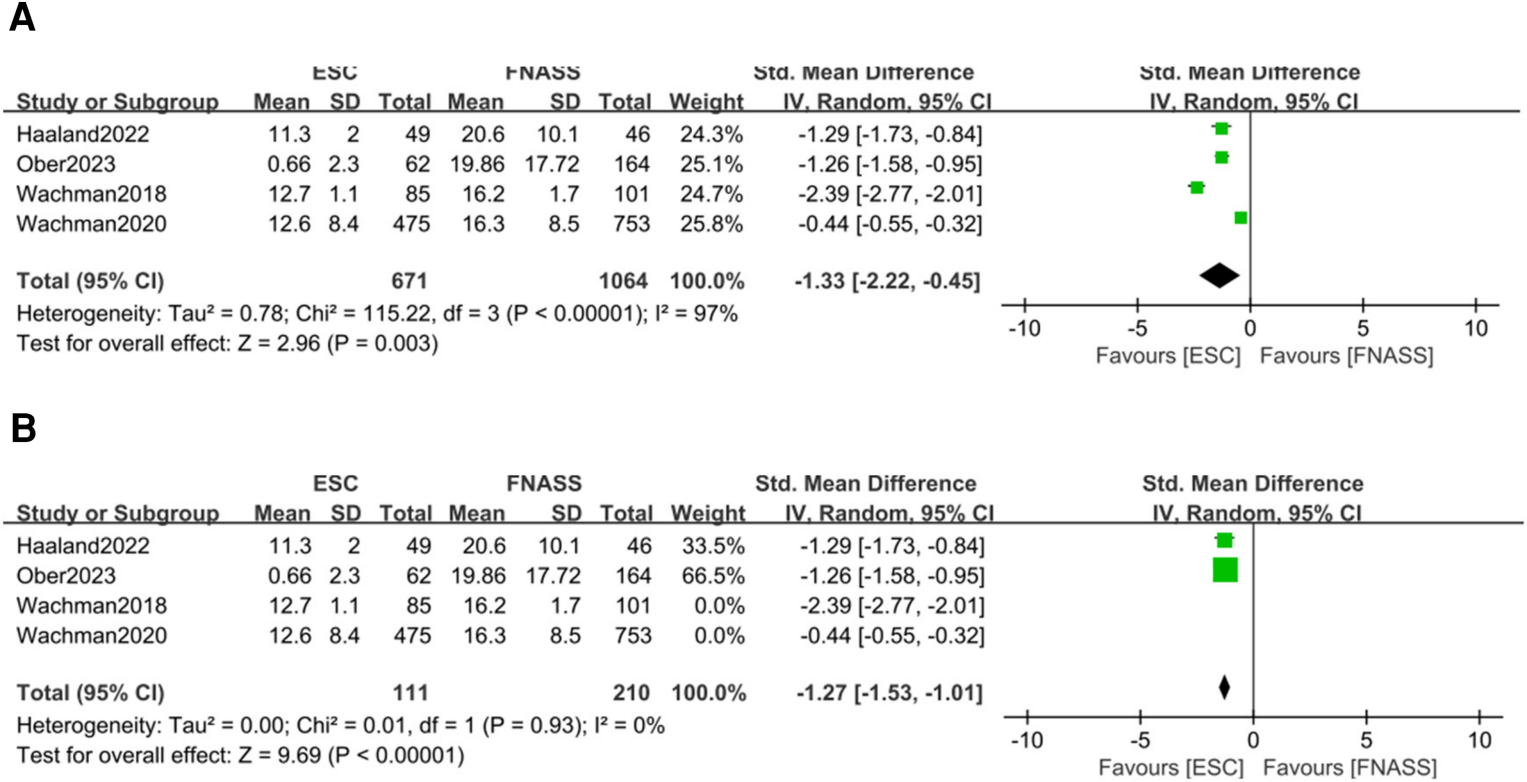
Eat, Sleep, Console model vs. Finnegan Neonatal Abstinence Scoring System for assessment on the length of opioid treatment stay. **(A)** Before sensitivity analysis. **(B)** After sensitivity analysis. SD, standard deviation; IV, inverse variance; CI, conﬁdence interval; ESC, Eat, Sleep, Console model; FNASS, Finnegan Neonatal Abstinence Scoring System.

Four studies (24, 30, 33, 37) indicated that infants with NOWS under ESC model had significantly shorter opioid treatment stays than those under the FNASS. The meta-analysis, depicted in Figure 6A, demonstrated superior results for the ESC model (SMD = −1.33, 95% CI = −2.22 to −0.45, *P = *0.003). Sensitivity analysis identified two studies (24, 30) as sources of high heterogeneity (*P *< 0.001, *I*^2^* *= 97%), but upon their exclusion, heterogeneity markedly decreased (*P *= 0.93, *I*^2^* *= 0%; Figure 6B). Given that the excluded studies were conducted by the same research team, it is plausible that the observed heterogeneity stemmed from differences in non-pharmacological treatment intervention methods.

## 4 Discussion

NOWS is characterized by symptoms that emerge following the abrupt cessation of maternally transferred opioids during the postpartum period (24, 39). Some studies have developed a novel, non-intrusive method for assessing newborns with NOWS, focusing on the infant's function rather than the severity of withdrawal symptoms (40). This approach enhances parental involvement by increasing opportunities for non-pharmacological therapy. Numerous studies (15, 17, 22, 24–38) have demonstrated that the use of the ESC model can significantly reduce both the length of hospital stays and the need for pharmacological treatment in infants with NOWS, thereby also diminishing postnatal pharmacological interventions (20). As hospitalization durations decrease, newborns can transition to the home environment more rapidly, facilitating their progression through normal developmental milestones such as sleep–wake cycles and tummy time.

As the ESC model gains prominence as a tool for evaluating NOWS, the body of related literature continues to grow. In this meta-analysis, all studies were retrospective and of medium to high quality, displaying consistent results across the dataset (Tables 2, 3). The risk of bias was considered low, given that all included studies adhered to a standardized definition of the ESC model and yielded objective results, such as the requirements for drug treatment, and the lengths of hospital and opioid treatment stays. However, the ESC model was not implemented in isolation. The included studies featured various co-interventions during the assessment, including changes in scoring practices that may account for some observed improvements. While it is possible that factors such as pharmacological treatment, skin-to-skin contact, and enhanced parental involvement could confound the results, we consider these not as confounding but as mediating factors that contribute to the benefits of the ESC model.

**Table 2:** Risk of bias for the randomized controlled trials.

Reference	Selection bias		Performance bias	Detection bias	Attrition bias	Reporting bias	Other bias	Total score
	Random sequence	Allocation hiding schemes	Whether to use the blind method	Result evaluator blind method	Whether the result data were complete	Whether the results were selectively reported	Other bias	
Young et al. (22)	High	Unclear	Unclear	Low	Low	Low	High	Moderate

**Table 3:** Risk of bias for the non-randomized trials.

Reference	Pre-intervention		At intervention	Post-intervention				Total score
	Confounding bias	Selection bias	Classification bias	Deviation bias	Missing data bias	Measurement of outcome bias	Selective reporting bias	Overall risk bias judgement
Grossman et al. (15)	Low	Low	Low	Low	Moderate	Low	Low	Moderate
Grossman et al. (17)	Low	Low	Low	Low	Moderate	Low	Low	Moderate
Wachman et al. (24)	Low	Low	Low	Low	Low	Low	Low	Low
Dodds et al. (25)	Low	No information	Low	Low	Moderate	Low	Low	Moderate
Achilles and Castaneda-Lovato (26)	Low	Low	Low	Low	Low	Low	Moderate	Moderate
Parlaman et al. (27)	Low	Low	Low	Low	Moderate	Low	Low	Moderate
Blount et al. (28)	Low	Low	Low	Low	Moderate	Low	Low	Moderate
Ponder et al. (29)	Low	Low	Low	Low	Low	Low	Low	Low
Wachman et al. (30)	Low	Low	Low	Low	Low	Low	Low	Low
Miller and Willier (31)	Low	Low	Low	Low	Moderate	Low	Low	Moderate
Ryan et al. (32)	Low	Low	Moderate	Low	Moderate	Low	Low	Moderate
Haaland et al. (33)	Low	Low	Low	Low	Low	Low	Low	Low
Amin et al. (34)	Low	Low	Low	Low	Moderate	Low	Low	Moderate
Chyi et al. (35)	Low	Low	Moderate	Low	Low	Low	Low	Moderate
Small et al. (36)	moderate	Low	Moderate	Low	Low	Low	Low	Moderate
Ober et al. (37)	Low	Low	Low	Low	Moderate	Low	Low	Moderate
Nicholson et al. (38)	Low	Low	Low	Low	Low	Low	Low	Low

**Table 4:** Characteristics of included studies.

	Reference	Year of study	Study design	No.	Intervention comparison	Outcomes	Data (ESC vs. FNASS)
1	Grossman et al. (15)	2017	Before-and-after assessment of QI	99	44 neonates: Eat, Sleep, Console model; 55 neonates: Finnegan Neonatal Abstinence Scoring System	Need for pharmacologic treatment (%)	14% vs. 98% *P* < 0.001
						Lengths of hospital stay, mean (SD)	5.9 (1.9) vs. 22.4 (10.8) *P* < 0.001
2	Grossman et al.	2018	Before-and-after assessment of QI	100	50 neonates: Eat, Sleep, Console model; 50 neonates: Finnegan Neonatal Abstinence Scoring System	Need for pharmacologic treatment (%)	12% vs. 62% *P* < 0.001
3	Wachman et al. (24)	2018	Before-and-after assessment of QI	186	85 neonates: Eat, Sleep, Console model; 101 neonates: Finnegan Neonatal Abstinence Scoring System	Need for pharmacologic treatment (%)	40% vs. 87% *P* < 0.0001
						Lengths of hospital stay, mean (SD)	11.3 (1.3) vs. 17.4 (1.6) *P *< 0.001
						Lengths of opioid treatment stay, mean (SD)	12.7 (1.1) vs. 16.2 (1.7) *P *= 0.0007
4	Dodds et al. (25)	2019	Before-and-after assessment of QI	82	33 neonates: Eat, Sleep, Console model; 49 neonates: Finnegan Neonatal Abstinence Scoring System	Need for pharmacologic treatment (%)	24% vs. 48% *P* < 0.05
						Lengths of hospital stay, mean (SD)	5.94 (2.98) vs. 11.77 (9.62) *P* < 0.05
5	Achilles and Castaneda-Lovato (26)	2019	Before-and-after assessment of QI	181	100: neonates Eat, Sleep, Console model; 81 neonates: Finnegan Neonatal Abstinence Scoring System	Need for pharmacologic treatment (%)	24% vs. 31% *P* < 0.001
						Lengths of hospital stay, mean (SD)	10.9 (2.7) vs. 18.7 (2.6) *P *= 0.005
6	Parlaman et al. (27)	2019	Before-and-after assessment of QI	304	155 neonates: Eat, Sleep, Console model; 149 neonates: Finnegan Neonatal Abstinence Scoring System	Need for pharmacologic treatment (%)	23% vs. 57% *P* < 0.05
						Lengths of hospital stay, mean (SD)	6.2 (1.2) vs. 9.0 (1.8) *P* < 0.05
7	Blount et al. (28)	2019	Before-and-after assessment of QI	76	36 neonates: Eat, Sleep, Console model; 40 neonates: Finnegan Neonatal Abstinence Scoring System	Need for pharmacologic treatment (%)	19% vs. 92% *P* < 0.05
						Lengths of hospital stay, mean (SD)	4.9 (1.8) vs. 10.3 (3.9) *P* < 0.05
8	Ponder et al. (29)	2020	Before-and-after assessment of QI	36	12 neonates: Eat, Sleep, Console model; 24 neonates: Finnegan Neonatal Abstinence Scoring System	Need for pharmacologic treatment (%)	58.3% vs. 91.7% *P *< 0.05
9	Wachman et al. (30)	2020	Before-and-after assessment of QI	1,228	475 neonates: Eat, Sleep, Console model; 753 neonates: Finnegan Neonatal Abstinence Scoring System	Need for pharmacologic treatment (%)	34.7% vs. 54.8% *P *< 0.001
						Lengths of hospital stay, mean (SD)	11.1 (7.9) vs. 14.2 (9.5) *P *< 0.001
						Lengths of opioid treatment stay, mean (SD)	12.6 (8.4) vs. 16.3 (8.5) *P *< 0.001
10	Miller and Willier (31)	2021	Before-and-after assessment of QI	71	37 neonates: Eat, Sleep, Console model; 34 neonates: Finnegan Neonatal Abstinence Scoring System	Need for pharmacologic treatment (%)	2.7% vs. 58% *P *< 0.05
11	Ryan et al. (32)	2021	Before-and-after assessment of QI	158	40 neonates: Eat, Sleep, Console model; 118 neonates: Finnegan Neonatal Abstinence Scoring System	Need for pharmacologic treatment (%)	33% vs. 53% *P *< 0.05
12	Haaland et al. (33)	2022	Before-and-after assessment of QI	95	49 neonates: Eat, Sleep, Console model; 46 neonates: Finnegan Neonatal Abstinence Scoring System	Need for pharmacologic treatment (%)	6.1% vs. 28.3% *P *< 0.05
						Lengths of hospital stay, mean (SD)	5.9 (3.1) vs. 8.4 (9.4) *P *= 0.08
						Lengths of opioid treatment stay, mean (SD)	11.3 (2.0) vs. 20.6 (10.1) *P *< 0.01
13	Amin et al. (34)	2023	Before-and-after assessment of QI	135	64 neonates: Eat, Sleep, Console model; 71 neonates: Finnegan Neonatal Abstinence Scoring System	Need for pharmacologic treatment (%)	27% vs. 34% *P *= 0.36
14	Chyi et al. (35)	2023	Before-and-after assessment of QI	251	100 neonates: Eat, Sleep, Console model; 151 neonates: Finnegan Neonatal Abstinence Scoring System	Need for pharmacologic treatment (%)	18% vs. 14.6% *P *= 0.47
15	Small et al. (36)	2023	Before-and-after assessment of QI	80	44 neonates: Eat, Sleep, Console model; 36 neonates: Finnegan Neonatal Abstinence Scoring System	Need for pharmacologic treatment (%)	0 vs. 47.2% *P *< 0.01
16	Young et al. (22)	2023	Before-and-after assessment of QI	1,305	603 neonates: Eat, Sleep, Console model; 702 neonates: Finnegan Neonatal Abstinence Scoring System	Need for pharmacologic treatment (%)	19.5% vs. 52% *P *< 0.05
						Lengths of hospital stay, mean (SD)	7.8 (0.7) vs. 14.0 (1.3) *P *< 0.05
17	Ober et al. (37)	2023	Before-and-after assessment of QI	226	62 neonates: Eat, Sleep, Console model; 164 neonates: Finnegan Neonatal Abstinence Scoring System	Lengths of hospital stay, mean (SD)	7.29 (2.6) vs. 24.8 (17.82) *P *< 0.001
						Lengths of opioid treatment stay, mean (SD)	0.66 (2.3) vs. 19.86 (17.72) *P *< 0.001
18	Nicholson et al. (38)	2023	Before-and-after assessment of QI	26	14 neonates: Eat, Sleep, Console model; 12 neonates: Finnegan Neonatal Abstinence Scoring System	Need for pharmacologic treatment (%)	7% vs. 58% *P *< 0.001

ESC, eat, sleep, console model; FNASS, Finnegan Neonatal Abstinence Scoring System; QI, quality intervention; M, mean; SD, standard deviation.

Although the ESC model is a relatively new approach for treating NOWS compared to the FNASS and conventional opioids, it has demonstrated significant reductions in pharmacological interventions and advantages in the evaluation and treatment of NOWS (15). The internal reliability of the FNASS necessitates repeated training, and errors or subjective assessments by scorers may lead to medical management decisions based on potentially inaccurate scores (14). The ESC model simplifies the evaluation process by prioritizing newborn function as the main measurement index, thus minimizing subjective influences (15, 17). When parameters are exceeded, the ESC model will administer opioids as needed but will not extend the treatment duration beyond a set period (18). Due to its novelty and the limited number of studies, further research is required to substantiate the ESC model's benefits. Despite advancements in evaluation and treatment methods, there is a scarcity of research on the long-term neurodevelopmental and behavioral outcomes of NOWS in all newborns. This gap may stem from stigma, as mothers, fearing the discovery of problems, might avoid developmental follow-up appointments, believing their child to be normal. This issue may also arise from several factors such as social barriers, communication, challenges, judgments by follow-up clinic staff, or loss of follow-up due to placement in child safety (15, 17, 22, 24–38). Regardless of the cause, this area of research warrants a comprehensive review of the various approaches for evaluating and managing NOWS.

## 5 Limitations

Some limitations of this study must be acknowledged. First, due to variations in health education, non-pharmacological treatment interventions, and data collection methods, a certain level of heterogeneity was present in this meta-analysis. Second, although genetic variation in opioid metabolism may influence infants’ responses to NOWS therapy, the impact of genetic factors remains unclear (41, 42). Third, this review did not address the short- and long-term consequences of inadequate treatment of neonatal neuromodulation dysfunction in the ESC model (43, 44). Furthermore, studies were included in the meta-analysis only if they provided the means and standard deviations of the relevant variables. Finally, since all research articles included in this review were in English, potential language bias may exist. Despite these limitations, our findings offer valuable insights for clinical practice.

## 6 Conclusions

Consistent evidence supports the ESC model as an effective tool for assessing and managing NOWS, significantly reducing the need for pharmacological therapy, the length of hospital stays, and the duration of opioid treatment. The ESC model enhances parental involvement in newborn care by increasing opportunities for non-pharmacological therapy, thereby facilitating a quicker transition of the newborn to the family environment. All studies included in this meta-analysis were retrospective, exhibiting low to moderate bias, and the findings were consistent across the studies. The ESC model is recommended as the preferred method for evaluating infants with NOWS.

## Data Availability

The original contributions presented in the study are included in the article/supplementary material, further inquiries can be directed to the corresponding author.
